# Genes Encoding Potential Molecular Mimicry Proteins as the Specific Targets for Detecting *Bursaphelenchus xylophilus* in PCR and Loop-Mediated Isothermal Amplification Assays

**DOI:** 10.3389/fpls.2022.890949

**Published:** 2022-05-11

**Authors:** Fanli Meng, Zhenkai Liu, Yongxia Li, Xingyao Zhang

**Affiliations:** ^1^The Key Laboratory for Silviculture and Conservation of Ministry of Education, College of Forestry, Beijing Forestry University, Beijing, China; ^2^Key Laboratory of Forest Protection of National Forestry and Grassland Administration, Ecology and Nature Conservation Institute, Chinese Academy of Forestry, Beijing, China; ^3^Co-Innovation Center for Sustainable Forestry in Southern China, Nanjing Forestry University, Nanjing, China

**Keywords:** *Bursaphelenchus xylophilus*, potential molecular mimicry proteins, specific target, rapid detection, PCR and LAMP

## Abstract

The introduction of the pine wood nematode (*Bursaphelenchus xylophilus*) to new areas has affected the international forestry industry because this pathogen causes pine wilt disease (PWD). Therefore, methods for the accurate and reliable detection of *B. xylophilus* are essential for controlling and managing this pest. The PCR and Loop-Mediated Isothermal Amplification (LAMP) techniques developed in this study involve species-specific primer sets targeting *B. xylophilus* genes encoding potential molecular mimicry proteins (*Bx-tlp-1*, *Bx-tlp-2*, and *Bx-cpi*), which are associated with pathogenicity. The PCR and LAMP results revealed that the primers were specific for *B. xylophilus Bx-tlp-1*, *Bx-tlp-2*, and *Bx-cpi*. Moreover, our LAMP assay targeting *Bx-tlp-1* conducted at 63°C detected *B. xylophilus* within 20 min and *B. xylophilus* from *Monochamus alternatus* or *M. saltuarius* within 30 min. The lower limits of detection for the LAMP and PCR assays were 10 pg and 10 ng genomic DNA, respectively, implying these assays may be useful for the rapid detection of *B. xylophilus* in pine forests. Designing primers specific for *Bx-tlp-1*, *Bx-tlp-2*, and *Bx-cpi* enabled the relatively rapid detection of *B. xylophilus* isolates as well as *M. alternatus* or *M. saltuarius* carrying *B. xylophilus*. These primers, which were designed following a thorough functional analysis of key *B. xylophilus* pathogenicity-related genes, may be useful for developing improved assays for the early diagnosis and prevention of PWD.

## Introduction

Pine is among the most popular timber species worldwide. Its ecological, economic, and social benefits are internationally recognized. Additionally, pine, which is an integral component of many products influencing human lifestyles, is a major forestation species in China (Jones et al., 2008; Soliman et al., 2012). However, it is susceptible to pine wilt disease (PWD) caused by the pine wood nematode *Bursaphelenchus xylophilus*,(Nickle et al., 1981), which poses a major threat to pine forests (Mamiya and Enda, 1972; Mota et al., 1999; Futai, 2013).

As a global forest quarantine disease, PWD can adversely affect forest ecologies and prevent sustainable timber production in the Americas, Europe, and Asia. *Bursaphelenchus xylophilus* is native to North America, but it was introduced to Japan in 1905, China in 1982, South Korea in 1988, Portugal in 1999, Spain in 2008, and the Madeira islands in 2010. After it was introduced to Europe, it spread to the Mediterranean coast (Futai, 2013). Since the first outbreak of PWD in China in 1982 (Cheng et al., 1983), the disease has been detected in 726 county-level administrative regions in 19 provinces. In the winter of 2017, PWD was detected in Fushun, Liaoning province, where the annual average temperature is 8.5°C referring to China Meteorological Administration. Almost 100,000 hectares were affected by PWD, resulting in the deaths of more than 50 million pine trees and economic losses exceeding 25 billion RMB. Hence, strengthening the quarantine and preventing the spread of PWD are the primary objectives of PWD disease management program.

The beetle species *Monochamus alternatus* and *M. saltuarius* are the principal vectors of *B. xylophilus* in Asia. These insects carry the dispersal fourth-stage juveniles of *B. xylophilus* and other nematodes (Ryss and Subbotin, 2017; Kanzaki and Giblin-Davis, 2018). Therefore, reliably discriminating *B. xylophilus* from other nematodes according to their morphological characteristics is a difficult task. Examining *M. alternatus* and *M. saltuarius* for the presence of *B. xylophilus* juveniles is an important part of an effective disease control strategy. More specifically, accurately identifying the juvenile stages is crucial for halting the spread of PWD.

Nematode species are routinely identified on the basis of their morphological characteristics. More specifically, *B. xylophilus* is typically identified according to the morphological characteristics of the adult-stage nematode; however, this requires a certain level of expertise and experience. Moreover, it is impossible to accurately identify the larvae. The limitations to morphology-based methods for identifying *B. xylophilus* have necessitated the development of methods relying on immunological, physiological, biochemical, and genetic characteristics. The current accepted methods for detecting and identifying *B. xylophilus* primarily involve molecular biology techniques. For example, the PCR(Wang et al., 2009) and loop-mediated isothermal amplification (LAMP) are commonly used detection techniques.

Regarding PCR, which enables the rapid amplification of DNA, two primers specific for the target DNA must be designed. The target sequence is then amplified by DNA polymerase (e.g., Taq DNA polymerase) in three steps (denaturation, renaturation, and extension). The amplified sequence serves as a template for the next cycle. Because each cycle takes 2–4 min to complete, the target sequence can be amplified several million times in 2–3 h. Because of it, the PCR technique is mature for clinical diagnoses and quarantine. The LAMP technique, which can be used to rapidly amplify DNA, requires four primers specific for six regions of the target DNA. The target sequence is then amplified by *Bst* DNA polymerase at a constant temperature between 60 and 65°C. As a rapid, simple, specific, sensitive, and low-cost technique, LAMP has been exploited for research regarding disease detection and gene chip development (Tomita et al., 2008).

In this study, we developed PCR- and LAMP-based methods for the direct detection of *B. xylophilus*. The proposed PCR and LAMP techniques use the species-specific primer sets targeting *B. xylophilus* genes encoding potential molecular mimicry proteins, including thaumatin-like protein-1 (*Bx-tlp-1*; accession number KM063438.1), thaumatin-like protein-2 (*Bx-tlp-2*; accession number MK000287), and a cysteine proteinase inhibitor (*Bx-cpi*; accession number MK000288). Shinya et al. (2013) performed a proteomic analysis and identified two putative *B. xylophilus* thaumatin-like proteins (TLPs) and one cysteine proteinase inhibitor with sequences that were highly similar to those of plant proteins. These proteins were subsequently determined to induce cell death in *Nicotiana benthamiana* (Kirino et al., 2020). Moreover, the TLP sequences in *B. xylophilus* and *Pinus massoniana* are reportedly similar (Wang et al., 2014). Additionally, Meng et al. (2017) cloned the gene encoding a *P. massoniana* TLP (*Pm-tlp*) and revealed that its expression is associated with the expression of the *B. xylophilus Bx-tlp-1*. The relatively high sequence similarity between potential molecular mimicry proteins and plant proteins suggests that they may have similar functions. An analysis of the expression of the potential molecular mimicry proteins in *B. xylophilus* infecting pine trees indicated that the temporal changes to the α-pinene content in the trees are consistent with the expression levels of the genes encoding a TLP (CPI) in *B. xylophilus* and *P. massoniana*. Thus, these genes are likely important for *B. xylophilus* infections of pine species (Meng et al., 2017, 2020).

Because the potential molecular mimicry proteins are associated with *B. xylophilus* pathogenicity, we designed primers specific for the *B. xylophilus* genome, which may be useful for developing new disease prevention and control measures.

## Materials and Methods

### Nematodes and Beetles

Both *B. xylophilus* and *Bursaphelenchus mucronatus* were raised on a *Botrytis cinerea* mycelial lawn on potato dextrose agar medium in plates at 25°C. The nematode cultures were stored in the laboratory. *Monochamus alternatus* and *M. saltuarius* were collected in different provinces in China, such as Fujian, Anhui, Zhejiang, Shandong, Guangdong, Tianjin, and Liaoning province. For each beetle, the thorax tissues were cut into two equal parts. One-half was used to isolate and observe nematodes by microscope, and the other half was used to extract DNA for detection, which was described as Meng et al. (2018).

### Genomic DNA Extraction

Genomic DNA was extracted from the nematode and beetle tissues as described by Meng et al. (2018) with some modifications. Single nematode was isolated from the culture medium, and transferred to a 200-μl Eppendorf tube. After adding 10 μl TE buffer, the tube was maintained in liquid nitrogen for 1 min and then incubated at 85°C for 1 min. This freeze–thaw treatment was performed three times. The supernatant was collected as the template nematode DNA sample. Beetle thorax tissues (0.2 g) were washed in sterilized water, centrifuged to remove as much water as possible, and then transferred to a 2-ml Eppendorf tube. After adding 200 μl TE buffer, the tube was placed in a grinder operated at 60 Hz for 5 min. After a centrifugation at 10,000 *g* for 1 min, the beetle samples were maintained in liquid nitrogen for 1 min and then incubated at 85°C for 1 min. This freeze–thaw treatment was performed three times, after which the samples were centrifuged at 10,000 *g* for 1 min. The supernatant was transferred to a 1.5-ml Eppendorf tube, and the freeze–thaw treatment and centrifugation were repeated. The supernatant was collected as the template beetle DNA sample.

### DNA Oligonucleotides

The PCR and LAMP primers designed and used in this study (Table 1) targeted *B. xylophilus* genes (*Bx-tlp-1*, *Bx-tlp-2*, and *Bx-cpi*) encoding previously identified potential molecular mimicry proteins (Meng et al., 2020). The PCR primers were designed using Primer 6.0. The six LAMP primers were designed using an online LAMP primer design program^1^ according to previously described criteria (Notomi et al., 2000). The primer set comprised two outer primers (F3 and B3), one forward inner primer (FIP), one reverse inner primer (BIP), and two loop primers (Loop F and Loop B). These primers, which anneal specifically to six distinct regions of the target DNA sequence, were synthesized by the Beijing Genomics Institute (Beijing, China). In addition, the LAMP primers targeting *syg-2* designed by Meng et al. (2018) are provided in the Supplementary Material (Supplementary Table S1).

**Table 1:** Sequences of the primers used to detect nematode and beetle DNA.

Test	Target	Primer name	Oligonucleotide sequence
PCR	*Bx-tlp-1*	tlp1-F	TGTGGCTGACACTTATGG
		tlp1-R	AGTCGTCGTTGTAGTTGATA
	*Bx-tlp-2*	tlp2-F	TCACACTTGCCGAGTTCTCCTTC
		tlp2-R	TCCGTGAGTCTTGCTATTGTCTCC
	*Bx-cpi*	cpi-F	CACGGCAAGTGCTAGGTGGATT
		cpi-R	TGAGCAGCGACAACTTGATGGAA
LAMP	*Bx-tlp-1*	tlp1F3	GCTGACACTTATGGTGGATAT
		tlp1B3	CCTCCGTGCTTGATCG
		tlp1FIP	AGTTGATAGTGAACTTGCTTAGCG+ATCGGATGTGCTATTGCC
		tlp1BIP	CGACGACTTCGTAGACACTTATGAA+CGCTGGTACAAGTCAAGTT
		tlp1LoopF	ACCGAGTGTGGTTCTTGAA
		tlp1LoopB	CAACGGCTATGACACTCCA
	*Bx-tlp-2*	tlp2F3	TGTGAAGCCTATACACTCCT
		tlp2B3	TCAGCAGAAGTACAAGTCG
		tlp2FIP	CCGCTCATAGCATCGTAAGTT+AATGATTACTATGACTTGAGCGT
		tlp2BIP	CGGATGCCTACCAGTATCC+GTGCCTCCAGTTCTCAC
		tlp2LoopF	CTGAAGACCGAAGCGTCAT
		tlp2LoopB	CGGAGACAATAGCAAGACTCA
	*Bx-cpi*	cpiF3	TCCTTGTCGGAGCCAA
		cpiB3	GCTTGACGATTTCGGTGA
		cpiFIP	TTGAGCAGCGAGCACTTGAT+CAAGAAGGCTGTCCACATT
		cpiBIP	TGGTGAGAACTACGCCATCG+TCATGCAACTGGATTACTTCTT
		cpiLoopF	CTGATGGCTTTGCTTCGC
		cpiLoopB	CGTAATTTGAGTCCGAATGTTACAA

### PCR and LAMP

The PCR mixture (25 μl) consisted of the following: PrimeSTAR HS DNA (Premix; TaKaRa, Japan), 12.5 μl; forward and reverse primers, 1 μl each; DNA template, 1 μl; and ddH_2_O, 9.5 μl. The PCR program was as follows: 94°C for 3 min; 35 cycles of 94°C for 30 s, 55°C for 30 s, and 72°C for 1 min; 72°C for 10 min. The amplified products were analyzed by agarose gel electrophoresis.

The LAMP mixture (25 μl) comprised the following: primers F3 and B3, 5 pmol each; primers FIP and BIP, 40 pmol each; primers Loop F and Loop B, 20 pmol each; 2 × Reaction Mix (Eiken Chemical, Japan), 12.5 μl; and DNA template, 1 μl. Samples were incubated at 63°C for 60 min and then at 85°C for 3 min to terminate the reaction. A positive control (purified *B. xylophilus* DNA) and a negative control (distilled water) were included in each run. The LAMP amplicons were detected by fluorescence using LightCycler 480 II system (Roche Diagnostics Ltd., Switzerland) or by a color change in the reaction mixture under natural light.

### Analysis of PCR and LAMP Assay Specificity and Sensitivity

To evaluate the specificity of the PCR and LAMP primer sets, the assays were performed using *M. alternatus* and *M. saltuarius* genomics DNA as well as the genomic DNA extracted from the following nematode, plant, and fungal species: *B. xylophilus*, *B. mucronatus*, *Bursaphelenchus fraudulentus*, *Bursaphelenchus conicaudatus*, *Bursaphelenchus corneolus*, *Bursaphelenchus firmae*, *Bursaphelenchus luxuriosae*, *Bursaphelenchus sexdentati*, *Aphelenchoides* sp., *Meloidogyne incongnita*, *Caenorhabditis elegans*, *Pinus thunbergii*, *Pinus massoniana*, *Helianthus annuus*, *Oryza brachyantha*, *Beauveria bassiana*, *Pochonia chlamydosporia*, *Penicillium griseofulvum*, *Paecilomyces lilacinus*, *Fusarium oxysporum* Schltdl, *B. cinerea*, and *Pestalotia diospyri* (Kikuchi et al., 2009; Ye and Giblin-Davis, 2013; Meng et al., 2020). At least three replicates were analyzed for each assay.

The sensitivity for the PCR and LAMP primer sets (i.e., minimal number of copies that could be detected) was assessed by performing the assays using a range of copy numbers (10^6^ to 10^1^) per reaction.

## Results

### PCR Detection of the Target Genes

**Figure 1:**
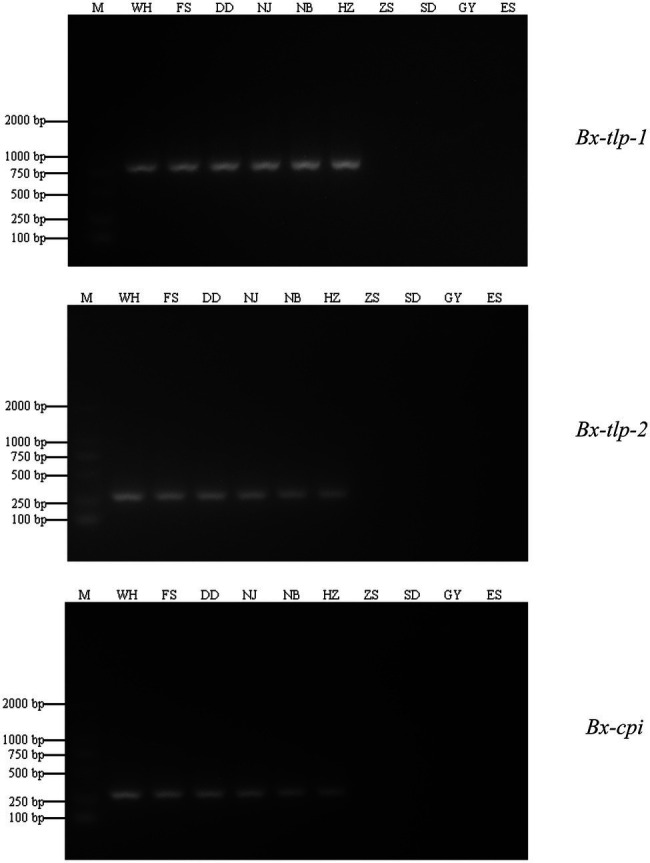
Specificity of the PCR primers for *Bursaphelenchus xylophilus* and *B. mucronatus* genes encoding potential molecular mimicry proteins. M, marker; WH, PCR amplification results for *B. xylophilus* from Shandong; NJ: the PCR amplification results for *B. xylophilus* from Jiangsu; NB: PCR amplification results for *B. xylophilus* from Zhejiang; HZ: PCR amplification results for *B. xylophilus* from Guangdong; FS and DD: PCR amplification results for *B. xylophilus* from Liaoning; ZS: PCR amplification results for *B. mucronatus* from Zhejiang; SD: PCR amplification results for *B. mucronatus* from Hunan; GY: PCR amplification results for *B. mucronatus* from Sichuan; ES: PCR amplification results for *B. mucronatus* from Hubei.

Fragments of the *B. xylophilus* genes encoding potential molecular mimicry proteins (*Bx-tlp-1*, *Bx-tlp-2*, and *Bx-cpi*) were amplified by PCR. The amplified *Bx-tlp-1*, *Bx-tlp-2*, and *Bx-cpi* fragments were 755, 241, and 202 bp, respectively (Figure 1). The sequences of these fragments are provided in the Supplementary Material (Supplementary Figures S1–S3).

### LAMP Detection of the Target Genes

**Figure 2:**
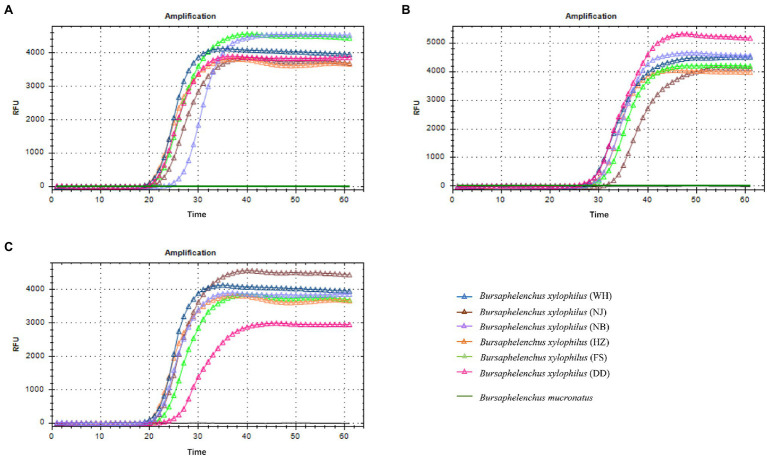
Specificity of the LAMP primers for *Bursaphelenchus xylophilus*. **(A)** *Bx-tlp-1* gene; **(B)** *Bx-tlp-2* gene; **(C)** *Bx-cpi* gene; WH: LAMP results for *B. xylophilus* from Shandong; NJ: LAMP results for *B. xylophilus* from Jiangsu; NB: LAMP results for *B. xylophilus* from Zhejiang; HZ: LAMP results for *B. xylophilus* from Guangdong; and FS and DD: LAMP results for *B. xylophilus* from Liaoning.

Amplified products are detectable in a LAMP assay within 60 min. In this study, the *Bx-tlp-1* and *Bx-cpi* LAMP amplicons were detectable within 20 min (Figures 2A,C), whereas the *Bx-tlp-2* LAMP amplicon was detectable within 30 min (Figure 2B).

### Specificity of the PCR and LAMP Primers for *Bursaphelenchus xylophilus*

Amplified products were obtained for the PCR analysis of genomic DNA extracted from the *B. xylophilus* isolates from Shandong, Jiangsu, Zhejiang, Guangdong, and Liaoning provinces. In contrast, PCR products were not generated for the other nematodes, plants, and fungi or for the negative control (i.e., no template). Accordingly, the PCR primers designed to target *Bx-tlp-1*, *Bx-tlp-2*, and *Bx-cpi* were specific for *B. xylophilus*. The LAMP primers targeting *syg-2* designed by Meng et al. (2018) were unable to produce detectable amplicons within 20 min for the *B. xylophilus* isolates from Liaoning province (FS and DD; Table 2). However, *Bx-tlp-1* and *Bx-cpi* LAMP amplicons were detectable within 20 min for all *B. xylophilus* isolates, suggesting the targeted *Bx-tlp-1* and *Bx-cpi* sequences may be specific to *B. xylophilus* (Supplementary Table S2; Supplementary Figure S4).

**Table 2:** Specificity of the PCR and Loop-Mediated Isothermal Amplification (LAMP) assays for *Bursaphelenchus xylophilus*.

	Code	Geographical origin	*syg-2* (Meng et al., 2018)				*Bx-tlp-1*				*Bx-tlp-2*				*Bx-cpi*			
			PCR	LAMP			PCR	LAMP			PCR	LAMP			PCR	LAMP		
				20 min	60 min	90 min		20 min	60 min	90 min		20 min	60 min	90 min		20 min	60 min	90 min
*Bursaphelenchus xylophilus*	WH	Shandong	+	+	+	+	+	+	+	+	+	−	+	+	+	+	+	+
*B. xylophilus*	NJ	Jiangsu	+	+	+	+	+	+	+	+	+	−	+	+	+	+	+	+
*B. xylophilus*	NB	Zhejiang	+	+	+	+	+	+	+	+	+	−	+	+	+	+	+	+
*B. xylophilus*	HZ	Guangdong	+	+	+	+	+	+	+	+	+	−	+	+	+	+	+	+
*B. xylophilus*	FS	Liaoning	+	−	+	+	+	+	+	+	+	−	+	+	+	+	+	+
*B. xylophilus*	DD	Liaoning	+	−	+	+	+	+	+	+	+	−	+	+	+	+	+	+
*Bursaphelenchus mucronatus*	ZS	Zhejiang	−	−	−	−	−	−	−	−	−	−	−	−	−	−	−	−
*B. mucronatus*	SD	Hunan	−	−	−	−	−	−	−	−	−	−	−	−	−	−	−	−
*B. mucronatus*	GY	Sichuan	−	−	−	−	−	−	−	−	−	−	−	−	−	−	−	−
*B. mucronatus*	ES	Hubei	−	−	−	−	−	−	−	−	−	−	−	−	−	−	−	−
*Bursaphelenchus fraudulentus*	BF	China	−	−	−	−	−	−	−	−	−	−	−	−	−	−	−	−
*Bursaphelenchus conicaudatus*	BC	China	−	−	−	−	−	−	−	−	−	−	−	−	−	−	−	−
*Bursaphelenchus corneolus*	BC2	China	−	−	−	−	−	−	−	−	−	−	−	−	−	−	−	−
*Bursaphelenchus firmae*	BF2	China	−	−	−	−	−	−	−	−	−	−	−	−	−	−	−	−
*Bursaphelenchus luxuriosae*	BL	China	−	−	−	−	−	−	−	−	−	−	−	−	−	−	−	−
*Bursaphelenchus sexdentati*	BS	China	−	−	−	−	−	−	−	−	−	−	−	−	−	−	−	−
*Aphelenchoides* sp.	ASP	China	−	−	−	−	−	−	−	−	−	−	−	−	−	−	−	−
*Meloidogyne incongnita*	MC	Beijing	−	−	−	−	−	−	−	−	−	−	−	−	−	−	−	−
*Caenorhabditis elegans*	CE	China	−	−	−	−	−	−	−	−	−	−	−	−	−	−	−	−
*Monochamus alternatus*	FZ	Fujian	−	−	−	−	−	−	−	−	−	−	−	−	−	−	−	−
*M. alternatus*	CZ	Anhui	−	−	−	−	−	−	−	−	−	−	−	−	−	−	−	−
*M. alternatus*	HZ	Zhejiang	−	−	−	−	−	−	−	−	−	−	−	−	−	−	−	−
*M. alternatus*	WD	Shandong	−	−	−	−	−	−	−	−	−	−	−	−	−	−	−	−
*M. alternatus*	HD	Guangdong	−	−	−	−	−	−	−	−	−	−	−	−	−	−	−	−
*Monochamus saltuarius*	FC	Liaoning	−	−	−	−	−	−	−	−	−	−	−	−	−	−	−	−
*M. saltuarius*	NZ	Liaoning	−	−	−	−	−	−	−	−	−	−	−	−	−	−	−	−
*M. saltuarius*	SM	Liaoning	−	−	−	−	−	−	−	−	−	−	−	−	−	−	−	−
*M. saltuarius*	YP	Liaoning	−	−	−	−	−	−	−	−	−	−	−	−	−	−	−	−
*M. saltuarius*	TJ	Tianjin	−	−	−	−	−	−	−	−	−	−	−	−	−	−	−	−
*Pinus thunbergii*	PT	Shandong	−	−	−	−	−	−	−	−	−	−	−	−	−	−	−	−
*Pinus massoniana*	PM	Zhejiang	−	−	−	−	−	−	−	−	−	−	−	−	−	−	−	−
*Helianthus annuus*	HA	Beijing	−	−	−	−	−	−	−	−	−	−	−	−	−	−	−	−
*Oryza brachyantha*	OB	China	−	−	−	−	−	−	−	−	−	−	−	−	−	−	−	−
*Beauveria bassiana*	BB	China	−	−	−	−	−	−	−	−	−	−	−	−	−	−	−	−
*Pochonia chlamydosporia*	PC	China	−	−	−	−	−	−	−	−	−	−	−	−	−	−	−	−
*Penicillium griseofulvum*	PG	China	−	−	−	−	−	−	−	−	−	−	−	−	−	−	−	−
*Paecilomyces lilacinus*	PL	China	−	−	−	−	−	−	−	−	−	−	−	−	−	−	−	−
*Oxysporum schltdl*.	OS	China	−	−	−	−	−	−	−	−	−	−	−	−	−	−	−	−
*Botrytis cinerea*	BC	China	−	−	−	−	−	−	−	−	−	−	−	−	−	−	−	−
*Pestalotia diospyri*	PD	China	−	−	−	−	−	−	−	−	−	−	−	−	−	−	−	−

+, amplified product; −, no amplified product.

### Sensitivity of the PCR and LAMP Assays

On the basis of a comparative analysis of the sensitivities of the two assays, we concluded that the detection limit of the fluorochrome dye used in the LAMP assay was 10 pg for the two positive results (i.e., for *Bx-tlp-1* and *Bx-cpi*), whereas the detection limit of the PCR assay for the same genes was 10 ng (Table 3). The LAMP primers used in this study to target the genes encoding potential molecular mimicry proteins were more sensitive than the primers designed by Meng et al. (2018), which were also associated with *B. xylophilus* pathogenicity.

**Table 3:** Comparison of the LAMP and PCR assay sensitivities.

Target genes	Test	Concentration of genomic DNA of *Bursaphelenchus xylophilus* after dilution					
		10^−1^	10^−2^	10^−3^	10^−4^	10^−5^	10^−6^
		10 ng	1 ng	100 pg	10 pg	1 pg	100 fg
*Bx-tlp-1*	LAMP	+	+	+	+	−	−
	PCR	+	−	−	−	−	−
*Bx-tlp-2*	LAMP	+	+	+	−	−	−
	PCR	+	−	−	−	−	−
*Bx-cpi*	LAMP	+	+	+	+	−	−
	PCR	+	−	−	−	−	−

+, amplified product; −, no amplified product.

### Utility of the PCR and LAMP Assays for Detecting *Bursaphelenchus xylophilus* From *Monochamus alternatus* or *Monochamus saltuarius*

**Figure 3:**
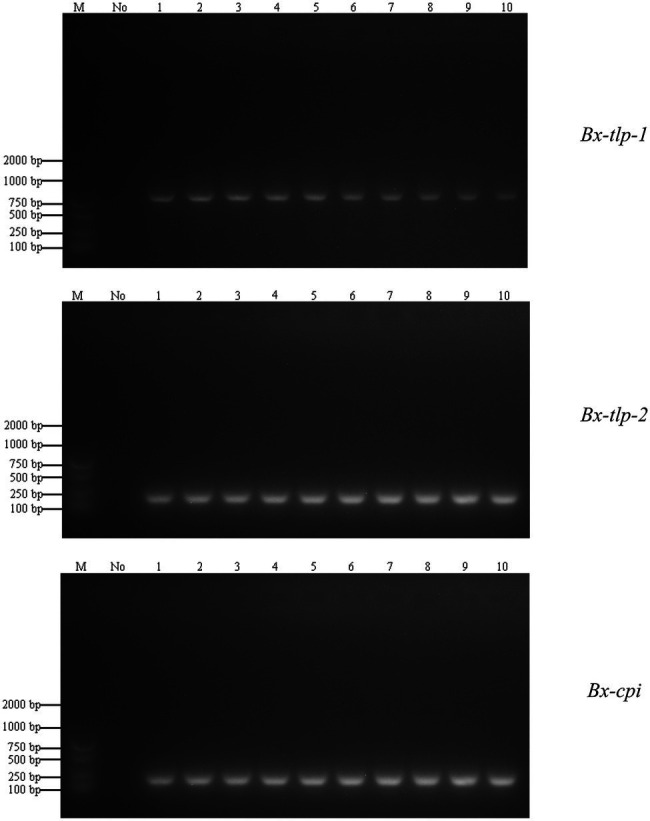
Specificity of the PCR primers for the genes encoding potential molecular mimicry proteins in *Bursaphelenchus xylophilus* from *Monochamus alternatus* or *M. saltuarius*. M: DNA marker AL2000; No: no *B. xylophilus* in *M. alternatus*; (1): PCR amplification results for *B. xylophilus* in *M. alternatus* from Fujian; (2): PCR amplification results for *B. xylophilus* in *M. alternatus* from Anhui; (3): PCR amplification results for *B. xylophilus* in *M. alternatus* from Zhejiang; (4): PCR amplification results for *B. xylophilus* in *M. alternatus* from Shandong; (5): PCR amplification results for *B. xylophilus* in *M. alternatus* from Guangdong; (6–9): PCR amplification results for *B. xylophilus* in *M. saltuarius* from Liaoning; and (10): PCR amplification results for *B. xylophilus* in *M. saltuarius* from Tianjin.

**Figure 4:**
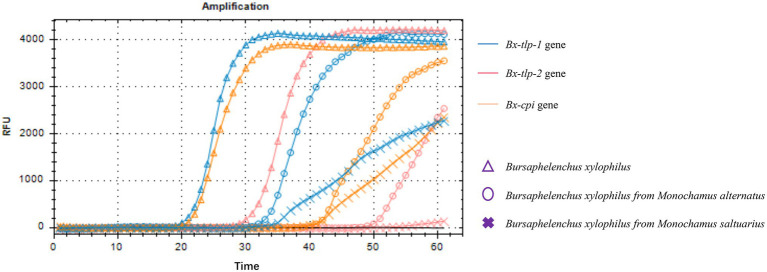
LAMP assay real-time monitoring using a light cycler and a fluorochrome dye.

The results of the PCR amplification conducted to detect *B. xylophilus* from *M. alternatus* or *M. saltuarius* collected from 10 regions in China are presented in Figure 3. Additionally, the LAMP assay results are presented as an amplification curve visualized using the fluorochrome dye. The results indicated that the primers targeting *Bx-tlp-1* and *Bx-cpi* were able to detect the genomic DNA of *B. xylophilus* from *M. alternatus* or *M. saltuarius* within 40 min, whereas the primers targeting *Bx-tlp-2* detected *B. xylophilus* genomic DNA within 50 min (Figure 4). The primers targeting *syg-2* (Meng et al., 2018) detected the genomic DNA of *B. xylophilus* from *M. saltuarius* within 50 min (Table 4). Hence, the primers designed for the genes encoding potential molecular mimicry proteins (*Bx-tlp-1*, *Bx-tlp-2*, and *Bx-cpi*) may be more sensitive than the primers targeting *syg-2*.

**Table 4:** LAMP-based detection of *Bursaphelenchus xylophilus* from *Monochamus alternatus* or *M. saltuarius*. +, LAMP amplicon detected; −, LAMP amplicon not detected.

Code	Vectors	Host	Geographical origin	*syg-2* (Meng et al., 2018)			*Bx-tlp-1*			*Bx-tlp-2*			*Bx-cpi*		
				30 min	40 min	50 min	30 min	40 min	50 min	30 min	40 min	50 min	30 min	40 min	50 min
1	*Monochamus alternatus*	*Pinus massoniana*	Fujian	−	+	+	+	+	+	−	−	+	−	+	+
2	*M. alternatus*	*P. massoniana*	Anhui	−	+	+	+	+	+	−	−	+	−	+	+
3	*M. alternatus*	*P. massoniana*	Zhejiang	−	+	+	+	+	+	−	−	+	−	+	+
4	*M. alternatus*	*P. massoniana*	Guangdong	−	+	+	+	+	+	−	−	+	−	+	+
5	*M. alternatus*	*P. thunbergii*	Shandong	−	+	+	+	+	+	−	−	+	−	+	+
6	*M. saltuarius*	*P. koraiensis*	Liaoning	−	−	+	+	+	+	−	−	+	−	+	+
7	*M. saltuarius*	*P. tabuliformis*	Liaoning	−	−	+	+	+	+	−	−	+	−	+	+
8	*M. saltuarius*	*P. tabuliformis*	Liaoning	−	−	+	+	+	+	−	−	+	−	+	+
9	*M. saltuarius*	*P. tabuliformis*	Liaoning	−	−	+	+	+	+	−	−	+	−	+	+
10	*M. saltuarius*	*P. tabuliformis*	Tianjin	−	−	+	+	+	+	−	−	+	−	+	+

## Discussion

The causative agent of PWD, *B. xylophilus*, is a prevalent organism carried by beetles (i.e., *M. alternatus* or *M. saltuarius*). Therefore, the numbers of beetles (*M. alternatus* or *M. saltuarius*) in a pine forest is a crucial indicator of the possibility of a PWD outbreak. Unless *B. xylophilus* from *M. alternatus* or *M. saltuarius* is specifically treated in the primary phase, the resulting PWD will often lead to tree death. The efficient and rapid detection of *B. xylophilus* from *M. alternatus* or *M. saltuarius* is necessary for preventing outbreaks *via* the implementation of specific treatments (Wang et al., 2009; Cardoso et al., 2012). Because death by PWD is very rapid, a novel method for detecting PWD must produce results quickly.

Proteins secreted by pathogens that are structurally or functionally similar to host defense-related proteins are called molecular mimicry proteins. Researchers identified substances released by pathogens that mimic plant defense-related compounds and disrupt physiologically important plant signaling pathways (Winn et al., 2021). Previous studies revealed the relatively high similarity between the Bx-TLP (Bx-CPI) and Pm-TLP (Pm-CPI) sequences, indicative of a similarity in their functions (Wang et al., 2014; Meng et al., 2017, 2020, 2022). Moreover, the expression of *B. xylophilus* genes encoding potential molecular mimicry proteins (*Bx-tlp-1*, *Bx-tlp-2*, and *Bx-cpi*) is responsive to α-pinene, which may affect terpene metabolism in pine trees and influence the pathogenicity of *B. xylophilus* (Meng et al., 2019, 2020, 2022).

In the present study, we designed specific primer sets targeting *Bx-tlp-1*, *Bx-tlp-2*, and *Bx-cpi* for the rapid detection of *B. xylophilus* and *M. alternatus* or *M. saltuarius* carrying *B. xylophilus*. The PCR and LAMP assays specifically detected all *B. xylophilus* isolates, with no amplification for all other examined species. The high specificity of the LAMP assay was conferred by the six primers targeting six regions of the *Bx-tlp-1* and *Bx-cpi* sequences, which were specific to *B. xylophilus*. The specificity of the LAMP primers used in this work was significantly better than that reported by Kikuchi et al. (2009; Supplementary Table S2), who detected *B. mucronatus* and *B. fraudulentus* in 90 min. On the other hand, there were no amplified products in other *Bursaphelenchus* group species, such as *B. firmae*, *B. luxuriosae*, and *B. sexdentati*. Meanwhile, the average concentration of genomic DNA from a single *B. xylophilus* was about 32 ng/μl (the volume was 10 μl), and the lower limits of detection for the LAMP and PCR assays were 10 pg and 10 ng genomic DNA, respectively. Moreover, LAMP primers targeting *Bx-tlp-1* or *Bx-cpi* were 5-fold more sensitive than the LAMP primers targeting *syg-2* (51.4 pg; Meng et al., 2018) and were able to rapidly detect *B. xylophilus* from *M. alternatus* or *M. saltuarius*. Thus, the key genes associated with *B. xylophilus* pathogenicity may be excellent targets for the rapid detection of the nematode because they may also be related to the fitness of the hosts, insects, and environment.

Considering the high sensitivity of the LAMP assay, the post-amplification procedures should be performed in a separate room (i.e., away from the PCR and LAMP reagents) to minimize the possibility of contamination (Kikuchi et al., 2009; Zhu et al., 2009; Meng et al., 2018). The LAMP assay requires only a regular water bath or a heat block that maintains the temperature at 63°C. Therefore, it is more cost-effective than a conventional PCR assay. Moreover, the two additional loop primers increase the speed and specificity of the amplification, resulting in faster reactions (relative to a conventional PCR). Additionally, the key genes associated with pathogenicity (e.g., those encoding potential molecular mimicry proteins) can be used as the specific targets for detection, which may be significant for preventing or controlling disease outbreaks. Our LAMP-based method detected *B. xylophilus Bx-tlp-1* within 20 min and *B. xylophilus* from *M. alternatus* or *M. saltuarius* within 30 min. These results are better than the LAMP assay results obtained by Meng et al. (2018) who detected the *B. xylophilus* target gene within 25 min and *B. xylophilus* from *M. alternatus* within 50 min. Furthermore, in contrast to a conventional PCR assay, the LAMP assay enables the detection of a positive amplification by the naked eye. The reactions and results can be interpreted simply by observing the color change in the reaction mixture. In addition to eliminating the need for the time-consuming electrophoretic analysis, LAMP assays can be performed without the sophisticated equipment needed for PCR (Kang et al., 2009; Huang et al., 2010; Hu et al., 2011; Kanetani et al., 2011; Meng et al., 2018).

In conclusion, *M. alternatus* is the main insect vector for *B. xylophilus*. The number of nematodes in *M. alternatus* is an important factor related to the distribution of *B. xylophilus*. Therefore, the fast and efficient detection of *M. alternatus* carrying *B. xylophilus* is necessary for the monitoring and early detection of PWD (Kikuchi et al., 2009; Hu et al., 2011; Cardoso et al., 2012; Meng et al., 2018). In this study, we designed PCR and LAMP primers specific for the *B. xylophilus* genes encoding potential molecular mimicry proteins (*Bx-tlp-1*, *Bx-tlp-2*, and *Bx-cpi*). The specific amplified fragments for *Bx-tlp-1*, *Bx-tlp-2*, and *Bx-cpi* were 755, 241, and 202 bp, respectively. However, no specific amplification products were detected for the other analyzed nematodes (e.g., *B. mucronatus*). These findings indicate that *Bx-tlp-1*, *Bx-tlp-2*, and *Bx-cpi* can be used to specifically detect *B. xylophilus* as well as *M. alternatus* or *M. saltuarius* carrying *B. xylophilus*. Furthermore, designing primers specific for *B. xylophilus* following an in-depth analysis of the functions of key pathogenicity genes may have important implications for future attempts at developing reliable methods for the early diagnosis and prevention of PWD.

## Data Availability Statement

The datasets presented in this study can be found in online repositories. The names of the repository/repositories and accession number(s) can be found in the article/Supplementary Material.

## Author Contributions

FM, YL, and XZ: experimental design. FM and ZL: experimental implementation. ZL: material contribution. FM: data analysis and manuscript writing. All authors contributed to the article and approved the submitted version.

## Funding

The present research was funded by the Fundamental Research Funds for the Central Universities (2021ZY03) and China Postdoctoral Science Foundation (2020M680410).

## Conflict of Interest

The authors declare that the research was conducted in the absence of any commercial or financial relationships that could be construed as a potential conflict of interest.

## Publisher’s Note

All claims expressed in this article are solely those of the authors and do not necessarily represent those of their affiliated organizations, or those of the publisher, the editors and the reviewers. Any product that may be evaluated in this article, or claim that may be made by its manufacturer, is not guaranteed or endorsed by the publisher.

## Footnotes

^1^ http://primerexplorer.jp/e/
