# Supplementary material for: Genes Encoding Potential Molecular Mimicry Proteins as the Specific Targets for Detecting Bursaphelenchus xylophilus in PCR and Loop-Mediated Isothermal Amplification Assays
Source: Front Plant Sci. 2022 May 11;13:890949. doi: 10.3389/fpls.2022.890949 (PMC9131030; doi:10.3389/fpls.2022.890949)
Supplement: Supplementary file 1 [file Data_Sheet_1.docx]

Table S1 Sequences of the LAMP primers designed by Meng *et al*.^17^

| Target | Primer Name | Oligonucleotide Sequence | Reference |
| --- | --- | --- | --- |
| *syg-2* | syg2-F3 | GTCTAACTTCTGCTGCCTATT | Meng, 2018 |
|  | syg2-B3 | CGATCTTGGCTTTCTCCC | Meng, 2018 |
|  | syg2-FIP | TTGTCAGCAAGTGAGTCTTCCC+GTCTCAGAGAACAAGCCTC | Meng, 2018 |
|  | syg2-BIP | AAGTACGGAGCGAAGATCACTT+AAGCGTCGGCTTCACATT | Meng, 2018 |
|  | syg2-LoopF | TGTCAAGCGGTCTAAGCG | Meng, 2018 |
|  | syg2-LoopB | AAGGACTCTGTGGTGCTTG | Meng, 2018 |

| Species | Code | Geographical Origin | Kikuchi *et al*. (2009) | | | *Bx-tlp-1* | | | *Bx-tlp-2* | | | *Bx-cpi* | | |
| --- | --- | --- | --- | --- | --- | --- | --- | --- | --- | --- | --- | --- | --- | --- |
|  |  |  | 20min | 60min | 90min | 20min | 60min | 90min | 20min | 60min | 90min | 20min | 60min | 90min |
| *Bursaphelenchus xylophilus* | WH | Shandong | － | ＋ | ＋ | ＋ | ＋ | ＋ | － | ＋ | ＋ | ＋ | ＋ | ＋ |
| *B. xylophilus* | NJ | Jiangsu | － | ＋ | ＋ | ＋ | ＋ | ＋ | － | ＋ | ＋ | ＋ | ＋ | ＋ |
| *B. xylophilus* | NB | Zhejiang | － | ＋ | ＋ | ＋ | ＋ | ＋ | － | ＋ | ＋ | ＋ | ＋ | ＋ |
| *B. xylophilus* | HZ | Guangdong | － | ＋ | ＋ | ＋ | ＋ | ＋ | － | ＋ | ＋ | ＋ | ＋ | ＋ |
| *B. xylophilus* | FS | Liaoning | － | ＋ | ＋ | ＋ | ＋ | ＋ | － | ＋ | ＋ | ＋ | ＋ | ＋ |
| *B. xylophilus* | DD | Liaoning | － | ＋ | ＋ | ＋ | ＋ | ＋ | － | ＋ | ＋ | ＋ | ＋ | ＋ |
| *Bursaphelenchus mucronatus* | ZS | Zhejiang | － | － | ＋ | － | － | － | － | － | － | － | － | － |
| *B. mucronatus* | SD | Hunan | － | － | ＋ | － | － | － | － | － | － | － | － | － |
| *B. mucronatus* | GY | Sichuan | － | － | ＋ | － | － | － | － | － | － | － | － | － |
| *B. mucronatus* | ES | Hubei | － | － | ＋ | － | － | － | － | － | － | － | － | － |
| *Bursaphelenchus fraudulentus* | BF | China | － | ＋ | ＋ | － | － | － | － | － | － | － | － | － |
| *Bursaphelenchus conicaudatus* | BC | China | － | － | － | － | － | － | － | － | － | － | － | － |
| *Bursaphelenchus* *corneolus* | BC2 | China | － | － | － | － | － | － | － | － | － | － | － | － |
| *Bursaphelenchus firmae* | BF2 | China | － | － | － | － | － | － | － | － | － | － | － | － |
| *Bursaphelenchus luxuriosae* | BL | China | － | － | － | － | － | － | － | － | － | － | － | － |
| *Bursaphelenchus sexdentati* | BS | China | － | － | － | － | － | － | － | － | － | － | － | － |
| *Aphelenchoides* sp*.* | ASP | China | － | － | － | － | － | － | － | － | － | － | － | － |
| *Meloidogyne incongnita* | MC | Beijing | － | － | － | － | － | － | － | － | － | － | － | － |
| *Caenorhabditis elegans* | CE | China | － | － | － | － | － | － | － | － | － | － | － | － |
| *Monochamus alternatus* | FZ | Fujian | － | － | － | － | － | － | － | － | － | － | － | － |
| *M. alternatus* | CZ | Anhui | － | － | － | － | － | － | － | － | － | － | － | － |
| *M. alternatus* | HZ | Zhejiang | － | － | － | － | － | － | － | － | － | － | － | － |
| *M. alternatus* | WD | Shandong | － | － | － | － | － | － | － | － | － | － | － | － |
| *M. alternatus* | HD | Guangdong | － | － | － | － | － | － | － | － | － | － | － | － |
| *Monochamus saltuarius* | FC | Liaoning | － | － | － | － | － | － | － | － | － | － | － | － |
| *M. saltuarius* | NZ | Liaoning | － | － | － | － | － | － | － | － | － | － | － | － |
| *M. saltuarius* | SM | Liaoning | － | － | － | － | － | － | － | － | － | － | － | － |
| *M. saltuarius* | YP | Liaoning | － | － | － | － | － | － | － | － | － | － | － | － |
| *M. saltuarius* | TJ | Tianjin | － | － | － | － | － | － | － | － | － | － | － | － |
| *Pinus thunbergii* | PT | Shandong | － | － | － | － | － | － | － | － | － | － | － | － |
| *Pinus massoniana* | PM | Zhejiang | － | － | － | － | － | － | － | － | － | － | － | － |
| *Helianthus annuus* | HA | Beijing | － | － | － | － | － | － | － | － | － | － | － | － |
| *Oryza brachyantha* | OB | China | － | － | － | － | － | － | － | － | － | － | － | － |
| *Beauveria bassiana* | BB | China | － | － | － | － | － | － | － | － | － | － | － | － |
| *Pochonia chlamydosporia* | PC | China | － | － | － | － | － | － | － | － | － | － | － | － |
| [*Penicillium griseofulvum*](http://www.hujiang.com/ciku/Penicillium_griseofulvum/) | PG | China | － | － | － | － | － | － | － | － | － | － | － | － |
| *Paecilomyces lilacinus* | PL | China | － | － | － | － | － | － | － | － | － | － | － | － |
| *Oxysporum schltdl*. | OS | China | － | － | － | － | － | － | － | － | － | － | － | － |
| *Botrytis cinerea* | BC | China | － | － | － | － | － | － | － | － | － | － | － | － |
| *Pestalotia diospyri* | PD | China | － | － | － | － | － | － | － | － | － | － | － | － |

Table S2 Specificity of the LAMP assays for *Bursaphelenchus xylophilus*. ＋, amplified product; －, no amplified product.

ATGAAGACCCTCATTCTTGCCGCTTTCGCTATTGCCGCTGTTGTTGCTGAC

ACTTATGGTGGATATACGAGCTTCGTTTTGAAGAATCAATGCAATCACGTG

F3

ATTTCTGTGTATCGGACAGGAAATAATCGGCCGGAGACGGACCAGTGCATC

CTGCCGACCGGTATCGGATGTGCTATTGCCTTCAAGAACCACACTCGGTTC

F2

GAGTTCAGGGCGAACAAGGAAGGCCGTGCGCTAAGCAAGTTCACTATCAAC

F1c

TACAACGACGACTTCGTAGACACTTATGAAATCGACGTCAGCAACGGCTAT

B1c

GACACTCCAGTTTCCATCCAACCCACCGATGGGAAATCGAAGAACTTGACT

B2

TGTACCAGCGCAACCTGCGCGGATGCCGGACAGAGCCAATCGATCAAGCAC

B3

GGAGGAAAATTCTACGTCATCTACTGTCCTTGATGTTAGTTAGTAGAAAAG

GCGTCCGCCGAGCGTCCGCCGACCATCCAGCGACTTTGCCAGGCCTCGTAG

GCACTTCCTACAATCTTCTTTCGGGTATCCACCTGCCCTACGCCTCGTATC

GATCGATTTTGTGTCGACCCGAACTGTCGTCGACCCGAAAAGTCGGCAAGC

GAAAAATTCTAGACGTCCGAAGGGCAAATTTGACATCATCAAATTTAATGT

TCTTTGCAAAATCCAATCCAAATTTGATCATTGAAACTCCCTATGAAAGAA

AAATATAAAATATTTTTTTAGGCCGTGCGCTAAGCAAGTTCACTAT

Figure S1 The amplified sequence of *Bx-tlp-1* fragment.

ATGAGTTGTGAAGCCTATACACTCCTCGCTTTAGGCCTCACACTTGCCGAG

F3

TTCTCCTTCGAGAATGGAGATGGTAATGATTACTATGACTTGAGCGTAATT

F2

GTTGGATTTGACGTGGGCATGACGCTTCGGTCTTCAGACGGCACAAACTTA

CGATGCTATGAGCGGGGCTGCCCGGATGCCTACCAGTATCCCGGAGACAAT

F1c B1c

AGCAAGACTCACGGAGTGAGAACTGGAGGCACGTTCGACTTGTACTTCTGC B2 B3

TGACAATAGCAAGACTCACGGA

Figure S2 The amplified sequence of *Bx-tlp-2* fragment.

ATGTTGTTCAAAGTTACTGTGCTCTTTGTTGTCTTGTTGGTTGCCCATCGC

TCCTTGTCGGAGCCAACCCAGAAGCCACGGCAAGTGCTAGGTGGATTTTCC

F3

GATGTCCCTTTGGATGATCCACTAGTTGTGAGGCTCGCCAAGAAGGCTGTC

F2

CACATTTTCGCGAAGCAAAGCCATCAGAAGCTAAAGTTCCATCAAGTGCTC

F1c

GCTGCTCAAAAACAAATTGTGAATGGTGAGAACTACGCCATCGATTTGGAG

B1c

GCCCGTAATTTGAGTCCGAATGTTACAAAAGAAGTAATCCAGTTGCATGAT

B2

TTTGTTCATGTTCCTTTGAAGGGCGGCAAAAACAGACATAACGTCACCGAA

B3

ATCGTCAAGCAAAAGTAA

Figure S3 The amplified sequence of *Bx-cpi* fragment.

Figure S4 Specificity of the LAMP primers for *Bursaphelenchus xylophilus*. A: *Bx-tlp-1* gene; B: *Bx-tlp-2* gene; C: *Bx-cpi* gene.
